# Editorial: Novel chemical, microbiological and physical approaches in food safety control, volume II

**DOI:** 10.3389/fnut.2024.1513864

**Published:** 2024-11-05

**Authors:** Marco Iammarino, Antonio Bevilacqua, Barbara Speranza, Sara Panseri, Gulhan Unlu, Rosalia Zianni

**Affiliations:** ^1^Chemistry Department, Istituto Zooprofilattico Sperimentale della Puglia e della Basilicata, Foggia, Italy; ^2^Department of Agriculture, Food, Natural Resources and Engineering, University of Foggia, Foggia, Italy; ^3^Department of Veterinary Medicine and Animal Sciences, University of Milan, Milan, Italy; ^4^Department of Animal, Veterinary and Food Sciences, University of Idaho, Moscow, ID, United States

**Keywords:** chemical analysis, food safety, microbiological analysis, novel approaches, physical analysis

The possible simultaneous presence of microbiological and chemical contaminants in the same food, also known as “cocktail effect” is an emerging topic in food safety. Given the presence of such cocktail of contaminants, high amounts of data are needed in order to develop any kind of evaluation, taking into account all possible interactions among different contaminants that can be hypothesized. Taking into account that the range of food which can be contaminated is very wide, and the need of comprehensive datasets for elaborating a significant statistical analysis, new effective analytical approaches are needed. These approaches can embrace new analytical procedures for chemical/physical determinations and new microbiological protocols characterized by high throughput, low cost and “Greenness” (1).

Most applications developed in the last years apply gas and liquid chromatography coupled to mass spectrometry for the quantification of a lot of food contaminants, detectable in the same run, using a single sample preparation procedure. Many approaches have been optimized for the determination of most significant concerns in food safety such as the presence of environmental contaminants, pesticides and veterinary drugs, etc. Moreover, the recent advances based on metabolomics, dark matter, protein fingerprint in microbiology, etc., open new frontiers in food analysis and food safety investigations.

Another new target of such novel methods is the “Green” aspects in terms of environmentally friendly processes to achieve a suitable sustainability (2).

As it regards microbiological approaches, a significant challenge in food safety is represented by the development of new and more effective methods for isolating and detecting most important foodborne pathogens, such as human noroviruses, O157:H7 and non-O157 STEC, etc. (3, 4).

This Research Topic was conceived in order to showcase the recent advances in food safety with respect to new chemical, microbiological and physical approaches for the determination and quantification of both chemical and microbiological risk factors that may contaminate food.

A brief overview of all articles collected in this Research Topic is reported below.

The original article proposed by Garcia et al. applied disk diffusion and microdilution methods for evaluating the antimicrobial and antibiofilm properties of *Boletus edulis* and *Neoboletus luridiformis* aqueous and methanolic extracts against ESKAPE isolates from clinical wound infections. High-performance liquid chromatography coupled to diode array detector was also used for phytochemical characterization based on the determination of total phenols, orthodiphenols and antioxidant activity. Finally, 3-(4, 5-dimethylthiazolyl-2)-2, 5-diphenyltetrazolium bromide (MTT) assay was performed to study the human foreskin fibroblasts-1 (HFF-1) cell viability. The authors concluded that the extracts of *B. edulis* and N. *luridiformis* exert antimicrobial and antibiofilm properties against multidrug-resistant bacteria with different efficacy rates. This effect can be explained by the relationship between the phenolic content/antioxidant activity and antimicrobial activity, especially due to the significant levels of protocatechuic acid, homogentisic acid, pyrogallol, gallic acid, p-catechin, and dihydroxybenzoic acid detected in the extract.

Luo et al. described a solid phase extraction coupled to high-performance liquid chromatography-tandem Orbitrap high resolution mass spectrometry (HPLC-Orbitrap HRMS) method for simple, sensitive, and rapid determination of 12 mycotoxins (ochratoxin A, ochratoxin B, aflatoxin B1, aflatoxin B2, aflatoxin G1, aflatoxin G2, HT-2 toxin, sterigmatocystin, diacetoxysciroenol, penicillic acid, mycophenolic acid, and citreoviridin) in edible oil, soy sauce, and bean sauce. This analytical method was characterized by low limits of detection and quantification, in the range 0.12–1.2 μg/L and 0.40–4.0 μg/L, respectively, recovery percentages in the range 78.3–115.6%, and relative standard deviations (RSDs) in the range 0.9–9.7%. The authors applied this method for the analysis of 24 samples of edible oil, soy sauce and bean sauce, verifying the presence of AFB1, AFB2, sterigmatocystin and mycophenolic acid in a concentration range from 1.0 to 22.1 μg/kg.

In the article by Yaman et al. three extraction solvents (water, methanol, and ethanol) were used to obtain soluble fractions of Turkish white cheese samples produced in a pilot plant scale. Then, reference methods, including gas and liquid chromatography, were used to detect and quantify amino acids, fatty acids and organic acids. FT-IR spectra were also collected and correlated with chromatographic data using pattern recognition analysis to develop regression and classification predictive models. The authors concluded that all models showed a good fit for predicting the target compounds during cheese ripening and that a simple methanolic extraction coupled to spectra obtained by a portable FT-IR device provides a fast, simple, and cost-effective technique to monitor the ripening of Turkish white cheese.

Wei-Ye et al. conducted a widely targeted metabolomics analysis of four commonly used edible and medicinal *Ganoderma* species based on ultra performance liquid chromatography-electrospray ionization-tandem mass spectrometry. The authors identified 575–764 significant differential metabolites among the species, most of which exhibited large fold differences. In particular, amino acids and derivatives, as well as terpenes, nucleotides and derivatives, alkaloids, and lipids resulted as the most advantageous metabolites of *Ganoderma lingzhi*. The authors also highlighted that the most significant metabolites in the four *Ganoderma* species may regulate and participate in signaling pathways associated with diverse cancers, Alzheimer's disease, and diabetes.

Guo et al. selected Pak choi (*Brassica rapa* L. ssp. *chinensis*) as a representative leafy vegetable to be tested in pots to reveal the effects of silicate–phosphate amendments on soil Cd chemical fractions, total plant Cd levels, and plant bioaccessibility. The authors used three heavy metal-immobilizing agents, namely wollastonite, potassium tripolyphosphate and sodium hexametaphosphate (SHMP). Using an *in vitro* digestion method combined with transmission electron microscopy, the authors reported that silicate and phosphorus agents were found to reduce the bioaccessibility of Cd in pak choi by up to 66.13%. The findings of this study contribute to the development of methods for safer cultivation of commonly consumed leafy vegetables and for soil remediation.

Marino et al. conducted a study on the nutritional and microbiological characteristics of *Aphia minuta*, a pelagic species harvested from the Golfo di Manfredonia in the Adriatic Sea (Italy). They assessed its chemical composition and profiles across different seasons, noting higher protein content in spring. Fatty acids analysis showed seasonal variations, with increased n-3 polyunsaturated fatty acids in spring and summer. Essential amino acids like leucine and lysine were most abundant in spring and summer, underlining nutritional value of this species. The research also examined the microbiological quality and shelf life of *Aphia minuta*, testing essential oils against bacteria and their effects in various packaging environments. Combining citrus extract with vacuum packaging notably reduced microbial counts within a week. The study offers valuable information to exploit the nutritional benefits and market potential of *Aphia minuta*.

The review prepared by Sajad et al. describes the vital role that pesticides play in modern agriculture by safeguarding crops from pests and diseases, despite concerns about their negative impact on human health and the environment. Pesticide residues in food and water sources pose significant health risks, potentially leading to cancer, endocrine disruptions, and neurotoxicity with prolonged exposure. To address these issues, researchers and health experts are seeking alternative strategies to counteract the harmful effects of pesticide residues. Nutraceuticals and bioactive compounds derived from whole foods like fruits, vegetables, herbs, and spices, are being explored for their potential to mitigate pesticide residue toxicity. These substances, comprising minerals, vitamins, antioxidants, and polyphenols, offer diverse biological actions that could aid in detoxification and recovery from pesticide exposure. The review aims to investigate the efficacy of nutraceutical interventions as a promising approach to combat the detrimental effects of pesticide residues.

The study presented by Chen et al. is focused on the photo-sensitivity of *Lyophyllum decastes*, an edible fungus, during mushroom emergence and its responses to various light qualities. *Lyophyllum decastes* was grown under different light conditions including white light, red light, blue light, mixed red and blue light, and a combination of far-red and blue light. Results indicated that red light led to negative effects on mycelium growth and primordial formation, while the combination of red and blue light significantly increased fruiting body characteristics. Blue light decreased stipe length and fruiting body weight. Blue and far-red light treatments deepened color in mushroom pileus and up-regulated blue photoreceptor genes. Fruiting bodies exposed to mixed red and blue light showed increased protein and polysaccharide content. The study offers insights for optimizing light conditions to enhance the quality of *Lyophyllum decastes* in industrial production.

Wei et al. investigated the Liuweizhiji Gegen-Sangshen (LGS), a very popular beverage in China for alleviating alcohol-related disorders and preventing alcoholic liver disease, in order to establish a comprehensive quality control methodology. Composed of six herbal components, LGS is polysaccharide-rich, yet the quality and activity of LGS-derived polysaccharides remained unexplored. Indeed, this study aimed to establish a quality control method for assessing LGS polysaccharides (LGSP) and investigate their antioxidant, anti-inflammatory, and prebiotic effects. LGSP was extracted and analyzed for molecular weight distribution, monosaccharide content, and structure applying various techniques such as high-performance size exclusion chromatography (HPSEC), 1-phenyl-3-methyl-5-pyrazolone-HPLC (PMP-HPLC), Fourier transform infrared spectroscopy (FT-IR) as well as nuclear magnetic resonance spectroscopy (NMR) techniques. Results revealed LGSP molecular weight distribution peaks, monosaccharide composition and structural characteristics. LGSP exhibited antioxidant properties, reduced pro-inflammatory factors, and enhanced the growth of beneficial probiotics. These findings contribute to understanding LGSP's structure-activity relationship, quality evaluation, and potential for developing healthy products.

Ingegno et al. developed and validated a highly sensitive analytical method based on gas chromatography–tandem mass spectrometry (GC–MS/MS) for the determination of polycyclic aromatic hydrocarbons (PAHs) in various baby foods. This study details the first setup of PAHs extraction and clean up through different QuEChERS-based methods and extraction solvents, considering factors like efficiency and recovery. The GC–MS/MS method was optimized, ensuring excellent linearity, sensitivity, and accuracy with detection limits ranging from 0.019 to 0.036 μg/kg. Recovery rates ranged from 73.1 to 110.7%, meeting European Commission guidelines. A survey on commercial infant food products of different types, based on meat, fish, legumes, and vegetables, confirmed PAH levels below quantification limits. The developed GC–MS/MS method offers a sensitive and reliable approach for PAH detection in baby foods, supporting in regulatory efforts for ensuring product safety and quality through regular monitoring.
